# Validation of the PCL-5 in Dutch trauma-exposed adults

**DOI:** 10.1186/s40359-024-01951-y

**Published:** 2024-08-28

**Authors:** Chris M. Hoeboer, Irina Karaban, Jeanet F. Karchoud, Miranda Olff, Mirjam van Zuiden

**Affiliations:** 1 Department of Psychiatry, Amsterdam UMC location University of Amsterdam, Meibergdreef 5, Amsterdam, 1005 AZ The Netherlands; 2 Amsterdam Public Health, Mental Health, Amsterdam, The Netherlands; 3 ARQ National Psychotrauma Centre, Diemen, The Netherlands; 4 Department of Clinical Psychology, Utrecht University, Utrecht, the Netherlands

## Abstract

**Background:**

The PTSD Checklist for DSM-5 (PCL-5) is an internationally widely used self-report questionnaire that can be used to screen for probable diagnosis of posttraumatic stress disorder (PTSD). Information on the psychometric properties of the Dutch PCL-5 is currently lacking.

**Objective:**

We aimed to validate the Dutch PCL-5 in a sample of Dutch adults with prior (suspected) serious injury and establish the optimal cut-off for probable PTSD diagnosis herein.

**Methods:**

Data for the current study were collected as part of a long-term follow-up measurement of the TraumaTIPS cohort, where adults admitted to an emergency department following (suspected) serious injury completed a follow-up measurement 12–15 years post-trauma. Of *N* = 333 eligible participants, *n* = 192 (57.7%) consented and completed the PCL-5 alongside self-report instruments measuring depression (QIDS), PTSD (IES-R), and quality of life (WHO-QOL and EQ-6D). In total, *n* = 185 participants also completed a clinician administered interview for PTSD (CAPS-5). Most participants were men (66%) and on average 54 years old (*SD* = 12.41). We evaluated the diagnostic utility of the PCL-5 using Youden index and tested reliability and convergent validity.

**Results:**

The PCL-5 demonstrated excellent diagnostic accuracy with a cut-off point of 16 resulting in an optimal Youden index (0.90) for screening purposes with a high sensitivity (1.00) and specificity (0.90). A cut-off of 22 yielded a slightly lower Youden index (0.84) but better positive predictive value (0.50 instead of 0.33) than the cut-off of 16. A cut-off of 29 resulted in the most accurate prevalence estimates. The PCL-5 showed a high internal consistency (Cronbach’s α = 0.94), excellent inter-item and item-total correlations and good convergent validity (*r* > .5 for CAPS-5, IES-R and QIDS).

**Conclusions:**

The PCL-5 is a reliable and valid measurement for PTSD symptoms and probable diagnosis and shows excellent screening abilities in Dutch adults with prior (suspected) serious injury, with a lower optimal cut-off compared to previously found in clinical populations. We recommend a cut-off of 22 for screening purposes and a cut-off of 29 for prevalence estimates in Dutch trauma-exposed adults.

**Supplementary Information:**

The online version contains supplementary material available at 10.1186/s40359-024-01951-y.

## Background

More than 80% of the individuals in the Netherlands experience at least one – and often multiple – potentially traumatic events throughout the course of their lives [1]. After exposure to a potentially traumatic event, many individuals initially experience post-traumatic symptoms such as intrusive memories about the event or sleeping problems. These symptoms usually subside within a few weeks, but for some they persist and develop into post-traumatic stress disorder (PTSD; [2]). The conditional risk of developing PTSD following a potentially traumatic event is on average 4.0%. The majority of the participants in the current study were admitted to the Emergency Department (ED) following road traffic accidents. Data from the World Health Organization mental health surveys indicate that exposure to automobile crashes and other life-threatening accidents are common potentially traumatic events, with 14.1% and 6.3% of the global population, respectively, experiencing such events at some point in their lives. The risk of developing PTSD related to automobile crashes is 2.1% and 5.1% for other life-threatening accidents. Hence, these events represent a significant public health concern [3]. PTSD includes intrusions about the traumatic event, avoidance of feelings and thoughts related to the event, negative alterations in mood and cognitions and alterations in arousal and reactivity [2]. PTSD represents a great burden both to the individual and society and is associated with many negative long-term outcomes, such as increased risk for morbidity and mortality, impaired work productivity and reduced quality of life [4–7]. When untreated, PTSD generally persists for many years [8], stressing the importance of identifying and treating individuals with PTSD.

Over the past decades, many studies found strong support for the clinical effectiveness and cost-effectiveness of trauma-focused treatments for PTSD [9–11]. However, most people with PTSD do not receive professional treatment [4, 12–14]. Even when patients seek treatment for their complaints, PTSD is often not recognized by physicians and therapists in routine clinical settings [14–17]. Reasons for this ‘treatment gap’ include limited available resources at mental healthcare institutions and a lack of knowledge about PTSD in healthcare in general [14]. Efficient and accurate screening tools are crucial to improve recognition of PTSD and overcome the treatment gap. Screening tools are usually filled out by trauma-exposed individuals themselves either online or via pencil-and-paper and therefore can be used in diverse settings without much effort or expert knowledge about PTSD required.

The PTSD Checklist for DSM-5 (PCL-5; [18]) is a 20-item self-report screening questionnaire that assesses the 20 DSM-5 diagnostic symptoms of PTSD and has been widely studied across the globe [19]. Research showed that the PCL-5 has an acceptable internal consistency, test-retest reliability and construct validity. However, most validation studies (> 65%) did not assess the actual diagnostic accuracy of the PCL-5 to determine a probable PTSD diagnosis, probably due to the time-consuming process of administering clinician administered interviews for PTSD [19]. Studies which assessed the diagnostic accuracy of the PCL-5 showed mixed results both regarding the optimal cut-off of the PCL-5 for determining a probable PTSD diagnosis (ranging from 22 to 49) and the performance of the PCL-5 with sensitivity ranging between 0.5 and 1, and specificity ranging between 0.35 and 0.97 [19]. Hence, there does not seem to be one general optimal cut-off point of the PCL-5 for determining probable PTSD. Despite the lack of information about the optimal cut-off points for the PCL-5 in many settings, it is still broadly used. Given the broad range of optimal cut-off points for the PCL-5, this might lead to many false positive or negative screenings. Therefore, recent studies have warned against overreliance on the PCL-5 as a measure of PTSD diagnostic status, especially when a used cut-off is not validated for the specific population and/or language of interest (e.g., [20]). The optimal cut-off varies across populations and languages, stressing the importance of establishing the diagnostic accuracy of the PCL-5 in various settings. Thus, more information about the performance and optimal cut-off of the PCL-5 across diverse populations is crucial.

Up to now the majority of the studies assessing the diagnostic accuracy of the PCL-5 included clinical *treatment-seeking* samples (usually from mental healthcare institutes), military personnel or student cohorts [19]. Only three out of the 64 validation studies (5%) included a trauma-exposed sample [21–23] and eight studies (12.5%) included community participants of which six included online samples recruited via Mturk [19]. This limits the generalizability of the results to very specific groups. Importantly, to reduce the treatment gap it is crucial that PTSD can also be effectively recognized in *trauma-exposed* individuals rather than treatment-seeking individuals. This is important since most trauma-exposed individuals with PTSD never seek treatment or their complaints are not correctly recognized by physicians precluding them from seeking help at a mental healthcare institute (e.g., [14]). Only one study assessed the general psychometric properties of the Dutch PCL-5 [24]. This study included 495 civilians after traumatic brain injury. The Dutch PCL-5 showed a high internal consistency and criterion validity. However, this study only relied on self-reported information and was therefore unable to test the screening ability of the Dutch PCL-5.

In addition to screening for probable PTSD on an individual level, the PCL-5 is often used to assess the prevalence of PTSD within a population. Recent meta-analyses summarizing PTSD prevalence estimates showed that screening instruments such as the PCL-5 are the most commonly used instruments for determining PTSD prevalence estimates [25–27]. In fact, only one meta-analysis was able to include a sufficient number of studies using clinician-assessed measures to investigate whether the type of assessment (screening versus clinician-assessed) moderated PTSD prevalence estimates [27]. Unsurprisingly, they found higher prevalence estimates for self-report compared to clinician-assessed instruments. Since the prevalence of PTSD in most populations is generally low compared to the prevalence of not having PTSD, a screening instrument with identical sensitivity and specificity will assign more false positives compared to false negatives. For example, with a PTSD prevalence of 10% and a sensitivity and specificity of 0.90, such screening will result in 1 false negative (10% of the 10 participants with PTSD) and 9 false positives (10% of the 90 participants without PTSD). When the optimal cut-off point is uncorrected for this imbalance, this will result in a large overestimation of the PTSD prevalence (in this example the prevalence estimate would be 18%, with 9 true positives and 9 false positives). Hence, it is important to separately provide optimal cut-off points of the PCL-5 for two purposes: (1) studies and/or clinical practice focused on identifying individuals with PTSD and (2) epidemiological studies into the prevalence of PTSD interested in the average prevalence across participants.

In the current study, we aimed to validate the PCL-5 in a sample of Dutch adults admitted to an emergency department (ED) following (suspected) serious injury 12–15 years ago and establish the diagnostic utility for screening on an individual level (i.e. optimal cut-off for probable PTSD diagnosis) and for determining prevalence estimates in this population. We also assessed the reliability and convergent validity of the Dutch PCL-5.

## Methods

### Participants

Data for the current study were collected as part of a long-term follow-up measurement of the TraumaTIPS cohort [28] 12–15 years after emergency department admission following (suspected) serious injury. The main aim of this long-term follow-up study was to determine long-term PTSD prevalence and associated adverse psychological, functional and economic outcomes within this sample. See Karchoud, Haagsma et al. [7] for the main outcome paper of this follow-up including information on the prevalence and associated psychological, functional and economic impact of long-term PTSD. All participants from the TraumaTIPS cohort were transported by ambulance or helicopter to the level-1 trauma centers of the Academic Medical Center (AMC) or VU University Medical Center (in Dutch ‘Vrije Universiteit Medisch Centrum’; VUmc) in Amsterdam, the Netherlands. Participants were presented with suspected severe injuries that required specialized acute medical care. Inclusion criteria of the TraumaTIPS cohort were: (a) age of 18 years or older; (b) proficiency in Dutch and (c) having experienced a potential traumatic event. Exclusion criteria were: (a) the injury resulting from deliberate self-harm; (b) suspected severe neurological conditions; (c) a psychotic disorder; (d) a bipolar disorder; (e) depressive disorder with psychotic features; (f) moderate to severe traumatic brain injury and (g) permanent residency outside the Netherlands. The follow-up assessment included no further in- or exclusion criteria, but specific inclusion (proficiency in Dutch) and exclusion (suspected severe neurological conditions clearly impairing cognition) criteria were checked again to ensure that participation was feasible. The follow-up assessment was exempted from formal review by the Medical Ethical Review Committee of the Amsterdam University Medical center (W20_035#20.063).

### Procedure

During their past participation, TraumaTIPS cohort study participants provided permission to contact them for follow-up measurements during the informed consent procedure. All original participants were contacted in the current study, except for those who withdrew their informed consent during the TraumaTIPS cohort study. We used existing contact details from 12 to 15 years ago, to contact eligible participants via telephone and email. When contact was established, we first verified their identification via a telephone conversation using personal details and by probing about their past index event in relation to their past research participation. After a positive ID- and contact details verification, study information about the TraumaTIPS long-term follow-up assessment was sent via email, including a link to the online informed consent form. Participants were asked to provide informed consent and thereafter fill out an online survey (consisting of several self-report questionnaires). Additionally, they completed the CAPS-5 interview online via a videocall-platform with a trained assessor. Participants were asked to complete the online survey before, or shortly after, partaking in the online CAPS-5 interview. See Karchoud, Haagsma [7] for more information on the current sample including flow diagram of recruitment process and comparison between completers and non-completers. In short, completers had significantly higher education levels (*p* < .001); less often a non-Dutch origin (*p* < .001); were more often in a committed relationship (*p* = .007); and reported lower PTSD symptom severity (*p* = .016) in the follow-up assessments of the original study compared to non-participants of the long-term follow-up.

### Measures

#### PCL-5

The PTSD checklist for DSM-5 (PCL-5) is a 20-item self-report questionnaire assessing probable PTSD diagnosis and PTSD symptom severity in the past month [29, 30]. For the current study, participants were instructed to keep the suspected severe injury in relation to their past research participation (i.e. their index event for research participation) in mind when filling out the PCL-5. During the PCL-5, participants are asked how much they have been bothered by each symptom over the past month. Every item is answered on a 5-point Likert scale ranging from not at all (0) to extremely [4]. The items can be categorized into four domains based on the DSM-5 with every item corresponding to a DSM-5 symptom: intrusions (five items), avoidance (two items), negative alterations in cognitions and mood (seven items) and hyperarousal (six items). Domain scores are calculated by summing the items of the domain. PCL-5 total score can be derived by summing the 20 items (range between 0 and 80) with higher scores indicating higher symptom severity. The Dutch translation of the PCL-5 has been established alongside the CAPS-5 translation [31] via an official translator and five senior psychotrauma experts who provided feedback on the translated PCL-5 in several rounds.

#### CAPS-5

The clinician-administered PTSD scale for DSM-5 (CAPS-5) is a clinician-assessed structured interview assessing PTSD diagnosis and symptom severity in the past month with 20 items [31, 32]. For the current study, participants were instructed to keep the suspected severe injury in relation to their past research participation (i.e. their index event for research participation) in mind during the CAPS-5 administration. Every item of the CAPS-5 is answered on a 5-point Likert scale ranging from not at all (0) to extremely [4]. For establishing a PTSD diagnosis, an item with a score of 2 or higher is considered endorsed. Every item of the CAPS-5 corresponds to the same DSM-5 symptom as the item of the PCL-5. A diagnosis of PTSD requires at least one clinically relevant intrusion item, at least one avoidance item, two negative alterations in cognitions and mood, two hyperarousal items and clinically significant distress or functional impairment. CAPS-5 total score can also be derived by summing the 20 items (range between 0 and 80) with higher scores indicating higher symptom severity. Previous validation studies in the United States and the Netherlands showed that the CAPS-5 PTSD diagnosis corresponded well with the CAPS-4 (*kappa* = 0.83), and CAPS-5 total scores showed a high internal consistency (α = 0.88) and test-retest reliability (ICC = 0.78; 31, 32). In the current study, the internal consistency of the CAPS-5 total score was high (α = 0.89). The CAPS-5 was administered by an assessor with a master’s degree in clinical psychology (IK) with previous extensive experience in CAPS-5 administration in the context of an international multi-center randomized clinical trial, who for the current study additionally completed several scoring exercises and reviewed mock CAPS-5 administrations and was supervised throughout the interview period. Every month a random selection of 15% of the audiotaped CAPS-5 measurements has been scored by a second researcher (independent of the original assessment) for interrater purposes. Both agreement on PTSD diagnoses (Cohen’s kappa = 1) and PTSD symptom severity (ICC = 0.80) was excellent.

#### IES-R

The impact of event scale: revised (IES-R) is a 22-item self-report questionnaire assessing PTSD symptom severity in the past week according to the DSM-IV [33]. The IES-R items do not refer to an index event. Every item is answered on a 5-point Likert scale ranging from not at all (0) to extremely [4]. The IES-R consists of three domains: intrusion, avoidance, and hyperarousal. Since the IES-R was not updated to match the DSM-5 PTSD criteria, it does not include the domain ‘negative alterations in cognitions and mood’. Moreover, the number of items per PTSD domain differs between DSM-IV and DSM-5. For example, the IES-R includes eight items tapping in avoidance instead of two items in the PCL-5 and CAPS-5. Total IES-R score can be derived by summing the 22 items (range between 0 and 88) with higher scores indicating higher symptom severity. Previous validation studies in the Netherlands showed that total IES-R scores from the Dutch version of the IES-R have a high internal consistency (α = 0.88) and showed adequate convergent validity [34]. In the current study, the internal consistency of the IES-R total score was high (α = 0.96).

#### QIDS-SR

The quick inventory of depressive symptomatology – self-reported (QIDS-SR) is a 16-item self-report questionnaire assessing symptoms for major depressive disorder in the past week according to the DSM-IV [35]. Items are answered on a 4-point Likert scale. The QIDS-SR consists of nine domains. Domain scores are based on the maximum score out of the items within the domain. Total QIDS-SR scores can be derived by summing the total domain scores (range between 0 and 27) with higher scores indicating higher symptom severity. A systematic review and meta-analysis from validation studies across the globe (including the Netherlands) showed that the QIDS-SR generally has an acceptable internal consistency (α > 0.65) and a moderate to high convergent validity [36]. In the current study, the internal consistency of the QIDS-SR total score was high (α = 0.80).

#### WHO-QOL

The World Health Organization Quality of Life –Abbreviation (WHO-QOL) is a 26-item self-report questionnaire assessing quality of life [37]. Items are answered on 5-point Likert scales. The WHO-QOL consists of four domains: physical health (PHYS), psychological (PSYCH), social relations (SOCIAL) and environment (ENVIR). Items within a domain can be summed and transformed on a scale from 0 to 100 with higher scores indicating better quality of life. A validation studies from the Netherlands showed that The WHO-QOL domains have an acceptable to good internal consistency (α ranging between 0.66 and 0.80) and a moderate to high convergent validity [38]. In the current study, the internal consistency of the WHO-QOL domains was moderate to high (α ranges between 0.72 and 0.87).

#### EQ-6D VAS scale

The EuroQol 6 dimensions Visual Analogue Scale (EQ-6D VAS scale) is one item assessing current health-related quality of life [39]. The item is scored on a scale from 0 (worst imaginable health) to 100 (best imaginable health). A systematic review summarizing validation studies of the EQ-6D (including the Netherlands) showed that the VAS scale demonstrates moderate to strong convergent validity [39].

#### LEC-5

The Life Events checklist for the DSM-5 (LEC-5; 18) is a 16-item self-report questionnaire assessing exposure to potentially traumatic events (PTEs). Each item represents one category of PTEs and participants are asked whether they experienced the event themselves, witnessed the event, encountered the event during work or learned about the event happening to a close friend or family member. For the current study, participants were asked to report on trauma exposure since the index trauma (suspected serious injury). A LEC-5 total score was calculated by summing all types of trauma exposure and all types of experiencing the event resulting in a possible range of 0–64 [40].

#### ISS and GCS

The Injury Severity Score (ISS; [41]) is an anatomical scoring system assessing the severity of injuries from patients with an overall severity score (range 0–75 with higher scores indicating more severe injuries). The Glasgow Coma Scale (GCS; [42]) (GCS) is a neurological scale assessing level of consciousness with three items: eye response (range 1–4), verbal response (range 1–5) and motor response (range 1–6). Total score can be calculated by summing all items (range 3–15) with higher scores indicating better consciousness.

### Statistical analyses

The statistical analysis plan of this study has been pre-registered prior to the analysis at the Open Science Framework (OSF; [43]). Analyses were performed using IBM SPSS Statistics Version 28.0 and R version 3.6.1 using package OptimalCutpoints [44].

For determining the diagnostic utility of the PCL-5 in screening for PTSD according to the CAPS-5 on an individual level, we used the Youden index which maximizes both sensitivity and specificity [45]. We used Receiver Operating Curve (ROC) analysis to determine the Area Under the Curve (AUC), sensitivity, specificity, positive predictive value (PPV), and negative predictive value (NPV) of the optimal cut-off point. The sensitivity, specificity, and Area Under The Curve (AUC) of the PCL-5 are satisfactory when > 0.75 for probable DSM-5 PTSD diagnosis according to CAPS-5 [46, 47]. For prevalence estimates, we provide the optimal cut-off score resulting in the most accurate prevalence estimates by balancing the number of false positives and false negatives. Note that this does not take any other screening performance indices into account and is only based on the cut-off resulting in the most accurate prevalence of PTSD on a group level.

To determine the reliability of the PCL-5, we calculated the Cronbach’s α of the PCL-5 total score and domain scores, and we assessed the corrected item-total (correlation between each item and the total score excluding that item) and inter-item correlations of the PCL-5 items. The Cronbach’s α of the PCL-5 total symptom score and domain scores is satisfactory when high (α ≥ 0.75), item total correlation is satisfactory when high (≥ 0.30), and inter-item correlation is satisfactory when moderate (between 0.15 and 0.50; [48]).

For determining the convergent validity of the PCL-5, we correlated the PCL-5 total score with theoretically similar constructs (CAPS-5 total score), related constructs (total QIDS-SR and IES-R scores) and theoretically opposite constructs (EQ-6D VAS score and WHO-QOL domain scores). We used the Spearman’s rho correlation coefficient because the PCL-5 total scores were not normally distributed. For similar constructs convergent validity is satisfactory when the correlation is high (r = ≥ 0.50, *p* < .05; 50). For related constructs, the convergent validity is satisfactory when the correlation is moderate (r = ≥ 0.30, *p* < .05). For theoretically opposite constructs, convergent validity is satisfactory when the correlation is negative and moderate (*r* ≤ − .30; [49]).

## Results

### Sample characteristics

Of the *N* = 333 eligible participants, *n* = 192 (57.7%) consented and completed all self-report measures in the current study. Of these, 185 participants also completed the CAPS-5 interview. Most participants were men (66%) and on average 54 years old (SD = 12.41). See Table 1 for characteristics of participants. Note that the Injury Severity Score (ISS) and Glasgow Come Scale (GCS) included missing data and were available for *n* = 157 (82% of the sample) and *n* = 147 (77% of the sample) respectively. PCL-5 total scores ranged between 0 and 70 (*M* = 6.83, *SD* = 10.41). CAPS-5 total scores ranged between 0 and 41 (*M* = 4.08, *SD* = 7.08). Nine participants out of the 185 (4.9%) met criteria for a PTSD diagnosis according to the CAPS-5. Table 2 lists the mean PCL-5 item scores for men and women separately.

**Table 1:** Characteristics of the participants

	No. (%)
Gender (women)	65 (34.2)
Age, mean (SD), years	54.47 (12.4)
Dutch country of origin	170 (91.4)
Married/cohabitating/committed relationship	160 (84.2)
Currently employed	122 (64.2)
Education, highest completed	
Primary education/high school/secondary education	51 (26.9)
Secondary vocational education	55 (28.9)
Higher vocational education or University	84 (44.2)
Has children / is a parent (yes)	143 (75.3)
Potentially traumatic event, type	
Traffic accident	127 (67.2)
Physical violence	4 (2.1)
Work-related accident	25 (13.2)
Fall	22 (11.6)
Other	11 (5.8)
	*M* (*SD*)
ISS overall severity score	9.68 (9.48)
GCS total score	14.21 (2.52)
LEC-5 total score	2.99 (2.80)

No. = number, SD = standard deviation, M = mean, ISS = Injury Severity Score, GCS = Glasgow Coma Scale, LEC-5 = Life Events checklist for the DSM-5

**Table 2:** PCL-5 item scores (mean and standard deviation) for men and women

	Women		Men	
PCL item	M	SD	M	SD
1 Intrusive memories	0.20	0.54	0.31	0.78
2 Repeated dreams	0.08	0.37	0.17	0.52
3 Reliving experience	0.08	0.27	0.17	0.52
4 Upset when reminded	0.28	0.76	0.22	0.69
5 Physical reaction when reminded	0.15	0.59	0.18	0.53
6 Avoiding internal reminders	0.22	0.65	0.25	0.62
7 Avoiding external reminders	0.25	0.56	0.18	0.61
8 Trouble remembering	0.88	1.23	0.52	1.10
9 Negative beliefs	0.09	0.29	0.26	0.77
10 Blaming yourself	0.42	0.92	0.53	0.93
11 Negative feelings	0.35	0.84	0.33	0.74
12 Loss of interest	0.34	0.69	0.44	0.91
13 Feeling distant	0.37	0.70	0.40	0.92
14 Trouble positive feelings	0.25	0.61	0.34	0.84
15 Irritable behavior	0.42	0.68	0.32	0.69
16 Risk taking	0.11	0.36	0.19	0.55
17 Being superalert	0.63	0.95	0.56	0.96
18 Feeling jumpy	0.48	0.83	0.25	0.70
19 Difficulty concentrating	0.71	1.00	0.63	1.06
20 Trouble sleeping	0.77	1.16	0.55	1.00

### Diagnostic utility

**Fig. 1:**
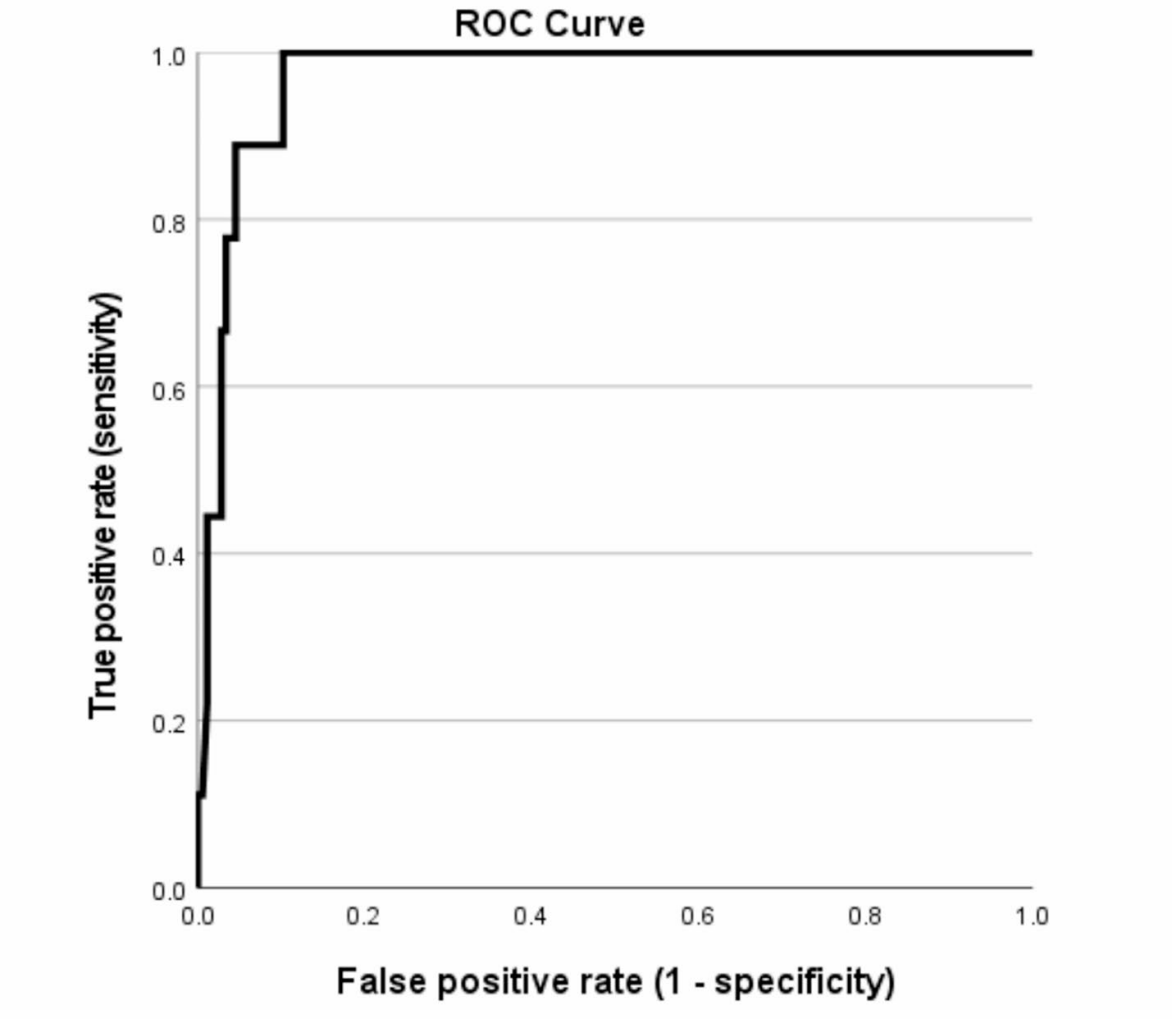
Receiver Operating Characteristics (ROC) curve of the PCL-5

The cut-off score with the highest Youden index of the PCL-5 for screening (on an individual level) for probable PTSD was 16. This cut-off yielded an excellent Youden’s index (0.90), sensitivity (1.00) and specificity (0.90). See Table 3 for more details and Fig. 1 for Receiver Operating Characteristics (ROC) curve. Hence, this cut-off resulted in 100% of the PTSD patients being correctly classified as having PTSD according to CAPS-5 and 90% of the patients without PTSD being correctly classified as not having PTSD. One-third of the people who screened positive were actually diagnosed with PTSD, while all people with a negative screening were not diagnosed with PTSD. The likelihood of having PTSD increases by almost 10 times (9.78) with a positive screening, while the likelihood of having PTSD with a negative screening is zero (no one with a negative screening actually had PTSD). Psychometric properties of all possible PCL-5 cut-offs are listed in Appendix 2. Note that a higher PCL-5 cut-off of 22 corresponds to a somewhat lower but still excellent Youden’s index (0.84), sensitivity (0.89) and specificity (0.95), and a higher positive predictive value (0.50) that the cut-off of 16 (0.33). Thus, a slightly lower sensitivity of this cut-off results in considerable higher chance of being a true positive when screening positive (50% instead of 33%). Therefore, this cut-off is recommended for screening purposes. An even higher cut-off would reduce the sensitivity considerably (to maximally 0.78 for a cut-off of 23) while the positive predictive value would only increase marginally (for the cut-off of 23 it is even decreased to 0.47 ; see Appendix 2).

For prevalence estimates, the optimal cut-off score of the PCL-5 was 29. This cut-off resulted in a reliable prevalence estimate of 5.41% in the current sample, close to the actual prevalence of 4.9% as determined using the CAPS-5.

**Table 3:** Cut-off points and performance of the PCL-5 for screening and prevalence estimates

	Screening	Screening	Prevalence estimates
Cut-off	16	22	29
AUC (95% CI)	0.97 (0.94 − 1.00)	0.97 (0.94 − 1.00)	0.97 (0.94 − 1.00)
Youden index	0.90	0.84	0.41
Prevalence estimate (%)	14.59	8.65	5.41
Sensitivity (95% CI)	1.00 (0.66 − 1.00)	0.89 (0.52 − 1.00)	0.44 (0.14-0.79)
Specificity (95% CI)	0.90 (0.84-0.94)	0.95 (0.91-0.98)	0.97 (0.93-0.99)
PPV (95% CI)	0.33 (0.23 − 1.00)	0.50 (0.33-0.98)	0.44 (0.25-0.79)
NPV (95% CI)	1.00 (0.97 − 1.00)	0.99 (0.96 − 1.00)	0.97 (0.87-0.99)
LR+ (95% CI)	9.78 (6.31–15.15)	19.56 (9.56–39.99)	15.64 (5.05–48.50)
LR- (95% CI)	0.00	0.12 (0.02-0.74)	0.57 (0.32-1.03)

AUC = Area Under The curve; CI = confidence interval; PPV = Positive Predictive Value; NPV = Negative Predictive Value; LR + = Positive Likelihood Ratio; LR- = Negative Likelihood Ratio

### Reliability

The PCL-5 total scores showed an excellent internal consistency (Cronbach’s α = 0.94). Cronbach’s α if item deleted indicated no possible improvements. In addition, PCL-5 domain intrusions (Cronbach’s α = 0.86), avoidance (Cronbach’s α = 0.79), negative alterations in cognitions and mood (Cronbach’s α = 0.85) and hyperarousal (Cronbach’s α = 0.86) all showed a good to excellent internal consistency. All corrected item-total correlations ranged between 0.34 and 0.78 with most (88%) inter-item correlations ranging between 0.15 and 0.60. PCL-5 item 8 (*trouble remembering*) was the only item with lower inter-item correlations (0.08 with item 1 and item 3). See Appendix 1 for complete inter-item correlation matrix.

### Convergent validity

Table 4 displays the correlation matrix between the PCL-5 total score and total scores of related constructs. The PCL-5 was strongly related to similar constructs with large correlation coefficients. Note that the correlation between the PCL-5 total score and some theoretically divergent constructs (WHO-social domain and EQ-6D vas scale) did not reach the predefined threshold of a moderate correlation.

**Table 4:** Spearman’s rho correlation matrix of PCL-5 total score with total scores of measures for convergent validity

	PCL-5	CAPS-5	IES-*R*	QIDS-SR	WHO-PHYS	WHO - PSYCH	WHO - SOCIAL	WHO-ENVIR	EQ-6D vas scale
PCL-5	1	0.65^*^	0.73^*^	0.53^*^	− 0.47^*^	− 0.46^*^	− 0.21^*^	− 0.33^*^	− 0.26^*^
CAPS-5		1	0.59^*^	0.50^*^	− 0.43^*^	− 0.40^*^	− 0.21^*^	− 0.19	− 0.24^*^
IES-R			1	0.53^*^	− 0.45^*^	− 0.37^*^	− 0.21	− 0.30^*^	− 0.24^*^
QIDS-SR				1	− 0.62^*^	− 0.56^*^	− 0.34^*^	− 0.35^*^	− 0.42^*^
WHO- PHYS					1	0.70^*^	0.55^*^	0.62^*^	0.57^*^
WHO - PSYCH						1	0.66^*^	0.66^*^	0.42^*^
WHO - SOCIAL							1	0.54^*^	0.38^*^
WHO-ENVIR								1	0.38^*^
EQ-6D vas scale									1

*Spearman’s rho correlation coefficient significant at 0.001 level; PCL-5 = PTSD Checklist for DSM-5; CAPS-5 = The clinician-administered PTSD scale for DSM-5; QIDS = Quick Inventory of Depressive symptomatology; IES-R = Impact of Events Scale-Revised; WHO-QOL = World Health Organization Quality Of Life; EQ-6D = EuroQol-6 Dimensions

## Discussion

The main aim of the present study was to validate the PCL-5 in a sample of Dutch adults admitted to an emergency department following (suspected) serious injury 12–15 years ago and more specifically to establish the performance of the PCL-5 in screening for PTSD within this sample. We found that the PCL-5 was an excellent screener for PTSD in this sample and demonstrated good psychometric properties in general. This implies that the PCL-5 can be effectively used to screen for PTSD on an individual level and for prevalence estimates in Dutch adults following exposure to (suspected) serious injuries.

We identified a PCL-5 total score of 16 or higher as cut-off with the best sensitivity and specificity for screening on an individual level for probable PTSD according to the CAPS-5. With this cut-off, all screening performance indices (sensitivity, specificity and AUC) of the PCL-5 were much better than the predefined minimum of 0.75. Nevertheless, we recommend a PCL-5 cut-off of 22 for screening purposes since this cut-off results in a higher chance of being a true positive when scoring above the cut-off while it still shows excellent performance indices. Since only about 1 out of 20 people in the current sample have PTSD, the number of false positives with a cut-off of 16 would be considerable. Therefore, the reduction in sensitivity with the threshold of 22 (and consequently additional missed patients with PTSD) seems worth the increase in chance that someone with a positive screening result actually has PTSD. This is especially relevant in settings where resources are limited or when it is not crucial that all true positives are correctly classified, for example in research. Notably, previous PCL-5 validation studies (all non-Dutch and usually including treatment-seeking samples) identified optimal cut-off scores of a PCL-5 total score between 23 and 49 [19], and as such the cut-offs we identified for Dutch individuals with prior (suspected) serious injury are somewhat lower. It has already been documented that the optimal cut-off of the PCL-5 varies due to contextual factors such as sample characteristics, cultural context and severity and prevalence of PTSD and comorbid conditions [19, 20]. The impact of the prevalence of the screening outcome within the investigated sample on the observed screening performance has been documented for decades across disciplines. This has been referred to as the *spectrum effect* [50, 51]. In the current study, we included a non-clinical trauma-exposed sample with a PTSD prevalence rate of 4.9% while previous studies often validated the PCL-5 in treatment seeking samples with much higher prevalence rates by definition [19]. Since many trauma-exposed individuals do not develop PTSD, prevalence rates and severity of PTSD are expected to be lower compared to treatment-seeking samples resulting in a relatively low optimal cut-off score. We also identified the optimal PCL-5 cut-off point for prevalence estimates. This is relevant for epidemiological studies interested in the point prevalence across participants rather than individual screening outcomes. Using the optimal cut-off point of 16 for screening on an individual level would result in an overestimation of the PTSD prevalence within the whole sample because most participants do not have PTSD (95%), resulting in more false positives than false negatives. We identified an optimal PCL-5 total score cut-off of 29 for PTSD prevalence estimates.

Our results imply that the Dutch PCL-5 can be effectively used to screen in Dutch individuals following exposure to (suspected) serious injuries both for clinical and research purposes. Note that the current sample includes a high percentage of male participants compared to the general Dutch society. The traumatic events presented at emergency departments are often from a physical rather than a sexual nature, while women more often experience sexual violence compared to men (e.g., [1, 13, 52, 53]). Thus, we expect that this is not problematic for the generalization of the current results to screening in emergency department settings, but results need to be replicated for screening in different settings, for example in victims of sexual violence or other interpersonal violence. Future research might also validate the PCL-5 in a Dutch treatment seeking sample to verify whether the low cut-off from the current study is indeed explained by the non-clinical sample or whether there is also a cultural element. Although the overall screening performance of the PCL-5 in the current study was excellent, the specificity was not perfect, thus resulting in false positives. These false positives may either be people without any mental health disorder or people with a mental health disorder with symptom overlapping with PTSD, such as depressive disorders. Such false positives have to be identified with follow-up clinician-administered interviews to avoid people receiving treatment which may not be needed or focusing on the wrong disorder.

Furthermore, we found that the Dutch PCL-5 is reliable, illustrated by excellent internal consistency, inter-item and corrected item-total correlations. This corresponds to previous PCL-5 validation studies [19]. We also found excellent convergent validity compared to clinical measures of PTSD and depression, but weaker relationships between PCL-5 total scores and measures of quality of life, especially the social relations domain. Most previous validation studies did not include a measure related to quality of life or social relations. One previous study included social support as measure of discriminant validity and found – similar to our finding – a modest relationship between PCL-5 total scores and social support [54]. Moreover, we found similarly modest relationships between other measures of PTSD (the CAPS-5 and DSM-IV based IES-R) and the social relations domain of quality of life in the current study so the modest convergent validity of the PCL-5 with quality of life does not seem to point towards problems with the PCL-5 specifically but more towards a generally weak relationship between PTSD and the social relations domain of quality of life. We conclude that overall the current data supports the convergent validity of the PCL-5.

Our study has several limitations. Firstly, the sample size was relatively small precluding investigation of the factor structure of the Dutch PCL-5, as is common in validation research. Secondly, this study was part of a long-term follow-up of trauma-exposed individuals and therefore the potentially traumatic event happened over a decade ago for all participants. About 5% of the respondents currently met criteria for PTSD related to this event which was comparable to the PTSD prevalence 12 months after the event (4.5%; 28). However the prevalence one month after the potentially traumatic event was higher (about 9%) and the optimal cut-off for probable PTSD might therefore be slightly different at this timepoint. More generally, research indicates that the prevalence of PTSD decreases until about 3–6 months following a potentially traumatic event [55]. Hence, we recommend to investigate the generalizability of the current findings for routine screening programs implemented within the first months after exposure to a potentially traumatic event. Thirdly, although the PCL-5 and CAPS-5 were specifically administered in relation to the index trauma of 12–15 years ago, we cannot exclude the possibility that previous or later trauma exposure nor psychiatric history affected the reported symptom severity. Fourthly, similar to past studies in individuals exposed to traumatic injuries [56] the current sample included predominantly men. It is currently still unclear how gender impacts screening performance and optimal cut-off scores [57, 58]. Future studies might focus on whether optimal cut-offs are similar for men and women. Fifthly, the current study predominantly included individuals with injuries resulting from non-interpersonal or non-intentional events, mostly traffic accidents. A previous PCL-5 validation study in the United States identified a higher optimal cut-off for probable PTSD in those with intentional injuries compared to non-intentional injuries [21]. Therefore, future studies might further investigate the optimal PCL-5 cutoffs for those with non-intentional injuries in the Netherlands. Moreover, research into the use of the PCL-5 for population who experienced other types of traumatic events is important such as sexual violence or death of a loved one. Lastly, as we did not include a retest measurement we are unable to provide information about the test-retest reliability of the Dutch PCL-5.

Despite the aforementioned limitations, the current study provides crucial information about the use of the Dutch PCL-5 in trauma-exposed individuals. We found that the Dutch PCL-5 is valid and reliable. Importantly, we found that the optimal cut-off for probable PTSD is relatively low in this trauma-exposed population compared to previous studies in usually help-seeking samples, but corresponds very well with actual PTSD diagnosis according to the CAPS-5. This implies that the PCL-5 can be effectively used to screen for PTSD in a sample of adults previously exposed to (suspected) serious injuries. More research into the optimal cut-off of the PCL-5 is important, especially in trauma-exposed populations where PTSD severity and prevalence rates are higher than the current sample. PTSD is often not recognized or misdiagnosed and therefore left untreated [14]. Screening for PTSD in a population following trauma exposure might be an important strategy to identify those with PTSD effectively so that they can receive evidence-based treatment.

### Electronic supplementary material

Below is the link to the electronic supplementary material.

Supplementary Material 1

## Data Availability

The preregistered data analysis plan, data file, syntax and outcome files are available on the Open Science Framework (OSF; https://osf.io/skfbq). We will document our data and code availability in the FAIR Traumatic Stress Data Sets library of the Global Collaboration on Traumatic Stress (GCTS).

## Abbreviations

PCL-5: PTSD Checklist for DSM-5
PTSD: Posttraumatic stress disorder
AMC: Academic Medical Center
VUmc: VU University Medical Center
CAPS-5: Clinician-administered PTSD scale for DSM-5
IES-R: The impact of event scale: revised
QIDS-SR: The quick inventory of depressive symptomatology – self-reported
WHO-QOL: The World Health Organization Quality of Life –Abbreviation
EQ-6D VAS scale: The EuroQol 6 dimensions Visual Analogue Scale
OSF: Open Science Framework
ROC: Receiver Operating Curve
AUC: Area Under the Curve
PPV: Positive predictive value
NPV: Negative predictive value
