# Supplementary material for: Validation of the PCL-5 in Dutch trauma-exposed adults
Source: BMC Psychol. 2024 Aug 28;12:456. doi: 10.1186/s40359-024-01951-y (PMC11351185; doi:10.1186/s40359-024-01951-y)
Supplement: Supplementary file 1 — Supplementary Material 1 [file 40359_2024_1951_MOESM1_ESM.docx]

| Appendix 1. Inter-item correlations for the PCL-5 | | | | | | | | | | | | | | | | | | | | |
| --- | --- | --- | --- | --- | --- | --- | --- | --- | --- | --- | --- | --- | --- | --- | --- | --- | --- | --- | --- | --- |
|  | item 1 | item 2 | item 3 | item 4 | item 5 | item 6 | item 7 | item 8 | item 9 | item 10 | item 11 | item 12 | item 13 | item 14 | item 15 | item 16 | item 17 | item 18 | item 19 | item 20 |
| item 1 | 1.000 | .538 | .715 | .655 | .539 | .620 | .509 | .077 | .514 | .290 | .502 | .482 | .461 | .515 | .413 | .405 | .534 | .469 | .419 | .486 |
| item 2 | .538 | 1.000 | .605 | .464 | .482 | .496 | .538 | .178 | .374 | .330 | .381 | .352 | .395 | .448 | .323 | .448 | .403 | .481 | .311 | .388 |
| item 3 | .715 | .605 | 1.000 | .504 | .528 | .577 | .467 | .076 | .519 | .233 | .506 | .453 | .415 | .546 | .390 | .399 | .509 | .506 | .350 | .440 |
| item 4 | .655 | .464 | .504 | 1.000 | .746 | .520 | .467 | .272 | .520 | .269 | .504 | .470 | .447 | .505 | .461 | .398 | .514 | .529 | .341 | .435 |
| item 5 | .539 | .482 | .528 | .746 | 1.000 | .454 | .446 | .272 | .528 | .141 | .417 | .380 | .373 | .440 | .363 | .310 | .484 | .469 | .252 | .338 |
| item 6 | .620 | .496 | .577 | .520 | .454 | 1.000+ | .649 | .315 | .570 | .448 | .557 | .490 | .475 | .449 | .469 | .423 | .531 | .462 | .402 | .493 |
| item 7 | .509 | .538 | .467 | .467 | .446 | .649 | 1.000 | .361 | .467 | .404 | .449 | .426 | .356 | .333 | .392 | .481 | .500 | .498 | .415 | .442 |
| item 8 | .077 | .178 | .076 | .272 | .272 | .315 | .361 | 1.000 | .216 | .369 | .291 | .211 | .253 | .174 | .327 | .234 | .214 | .185 | .184 | .298 |
| item 9 | .514 | .374 | .519 | .520 | .528 | .570 | .467 | .216 | 1.000 | .347 | .623 | .604 | .674 | .622 | .527 | .458 | .579 | .501 | .485 | .522 |
| item 10 | .290 | .330 | .233 | .269 | .141 | .448 | .404 | .369 | .347 | 1.000 | .407 | .398 | .406 | .329 | .295 | .185 | .382 | .257 | .364 | .341 |
| item 11 | .502 | .381 | .506 | .504 | .417 | .557 | .449 | .291 | .623 | .407 | 1.000 | .694 | .747 | .730 | .634 | .468 | .573 | .529 | .595 | .544 |
| item 12 | .482 | .352 | .453 | .470 | .380 | .490 | .426 | .211 | .604 | .398 | .694 | 1.000 | .752 | .661 | .576 | .376 | .554 | .516 | .645 | .546 |
| item 13 | .461 | .395 | .415 | .447 | .373 | .475 | .356 | .253 | .674 | .406 | .747 | .752 | 1.000 | .754 | .682 | .469 | .517 | .588 | .659 | .589 |
| item 14 | .515 | .448 | .546 | .505 | .440 | .449 | .333 | .174 | .622 | .329 | .730 | .661 | .754 | 1.000 | .501 | .451 | .579 | .606 | .563 | .512 |
| item 15 | .413 | .323 | .390 | .461 | .363 | .469 | .392 | .327 | .527 | .295 | .634 | .576 | .682 | .501 | 1.000 | .579 | .452 | .522 | .652 | .626 |
| item 16 | .405 | .448 | .399 | .398 | .310 | .423 | .481 | .234 | .458 | .185 | .468 | .376 | .469 | .451 | .579 | 1.000 | .377 | .454 | .431 | .402 |
| item 17 | .534 | .403 | .509 | .514 | .484 | .531 | .500 | .214 | .579 | .382 | .573 | .554 | .517 | .579 | .452 | .377 | 1.000 | .703 | .488 | .523 |
| item 18 | .469 | .481 | .506 | .529 | .469 | .462 | .498 | .185 | .501 | .257 | .529 | .516 | .588 | .606 | .522 | .454 | .703 | 1.000 | .529 | .505 |
| item 19 | .419 | .311 | .350 | .341 | .252 | .402 | .415 | .184 | .485 | .364 | .595 | .645 | .659 | .563 | .652 | .431 | .488 | .529 | 1.000 | .662 |
| item 20 | .486 | .388 | .440 | .435 | .338 | .493 | .442 | .298 | .522 | .341 | .544 | .546 | .589 | .512 | .626 | .402 | .523 | .505 | .662 | 1.000 |

Appendix 2. Performance indices of all possible PCL-5 cut-offs

| PCL-5 cut-off | Youden index | Sensitivity | Specificity | PPV | NPV |
| --- | --- | --- | --- | --- | --- |
| 1 | .28 | 1 | .28 | .07 | 1.00 |
| 2 | .37 | 1 | .37 | .08 | 1.00 |
| 3 | .47 | 1 | .47 | .09 | 1.00 |
| 4 | .61 | 1 | .61 | .12 | 1.00 |
| 5 | .65 | 1 | .65 | .13 | 1.00 |
| 6 | .70 | 1 | .70 | .15 | 1.00 |
| 7 | .73 | 1 | .73 | .16 | 1.00 |
| 8 | .77 | 1 | .77 | .18 | 1.00 |
| 9 | .78 | 1 | .78 | .19 | 1.00 |
| 10 | .81 | 1 | .81 | .21 | 1.00 |
| 11 | .83 | 1 | .83 | .23 | 1.00 |
| 12 | .85 | 1 | .85 | .26 | 1.00 |
| 13 | .86 | 1 | .86 | .27 | 1.00 |
| 14 | .88 | 1 | .88 | .29 | 1.00 |
| 15 | .88 | 1 | .88 | .30 | 1.00 |
| 16 | .90 | 1 | .90 | .33 | 1.00 |
| 17 | .79 | .89 | .90 | .31 | .99 |
| 18 | .82 | .89 | .93 | .38 | .99 |
| 19 | .83 | .89 | .94 | .42 | .99 |
| 20 | .83 | .89 | .94 | .44 | .99 |
| 21 | .84 | .89 | .95 | .47 | .99 |
| 22 | .84 | .89 | .95 | .50 | .99 |
| 23 | .73 | .78 | .95 | .47 | .99 |
| 25 | .74 | .78 | .96 | .50 | .99 |
| 26 | .75 | .78 | .97 | .54 | .99 |
| 27 | .64 | .67 | .97 | .50 | .98 |
| 28 | .64 | .67 | .97 | .55 | .98 |
| 29 | .41 | .44 | .97 | .44 | .97 |
| 30 | .42 | .44 | .98 | .50 | .97 |
| 33 | .43 | .44 | .99 | .67 | .97 |
| 36 | .32 | .33 | .99 | .60 | .97 |
| 39 | .21 | .22 | .99 | .50 | .96 |
| 43 | .10 | .11 | .99 | .50 | .96 |
| 70 | .11 | .11 | 1 | 1 | .96 |
